# Case Series: Episodes of Care in an Intensive Care Garden—Does Fresh Air Matter?

**DOI:** 10.1111/nicc.70508

**Published:** 2026-07-01

**Authors:** Kate Tantam, Susie Wolstenholme, Johnny Scott, Jude Fewings, Genevieve Conquest

**Affiliations:** ^1^ Intensive Care Unit University Hospitals Plymouth NHS Trust Plymouth UK; ^2^ Plymouth University Plymouth UK

## Abstract

**Background:**

Access to nature is found to have significant mental and physical health benefits, but there is minimal evidence of the potential impact on patients, staff and relatives from intensive care.

**Aims:**

It is the purpose of this paper to explore a case series of 2 patients from an intensive care in the United Kingdom (UK), utilising a garden space for end‐of‐life care and rehabilitation practices.

**Study Design:**

In the first case, care at end‐of‐life is explored from a relative and staff perspective. The second case explored the impact of utilisation of a garden space for the care of a patient with multi‐drug‐resistant organisms in intensive care.

**Results:**

Feedback from the first case explored that, despite the sadness of the end‐of‐life experience, the intensive care garden space provided a positive environment for all respondents. Four main themes emerged: environmental influence, personalisation and privacy, communication and perception of care of the patient and relative. The second case explored the positive impact of environmental conditions to control and manage the spread of complex drug‐resistant pathogens, and the role of the interdisciplinary team to facilitate the management of complex delirium.

**Conclusion:**

The utilisation of a garden room and fresh air space in both cases was found to optimise patient, relative, staff experience and in case 2 it was used successfully to support delirium management and rehabilitation experience.

**Relevance to Clinical Practice:**

Utilisation of a nature‐based environment can impact positively patient, relative and staff experience of critical care, with careful attention to risk assessment and safety. However more clinical research is required to further explore the phenomenon.

## Impact Statements

- What is known about the topic
  ○ Nature exposure has positive mental and physical health benefits.
  ○ The physical environment in ICU can negatively impact patient, visitors to ICU and staff experience.
  ○ Little empirical evidence is available evaluating nature exposure in the ICU patient population.
- What this paper adds
  ○ Nature exposure can positively influence the ICU experience at end‐of‐life.
  ○ Nature exposure can be facilitated to support rehabilitation of complex patients.
  ○ Further research is required to fully explore the impact of nature interventions for ICU patients.

*Second only to air is light as an essential for growth, health and recovery from sickness—not only daylight, but sunlight—and indeed fresh air must be sun‐warmed, sun‐penetrated air. This should be meant to include colour, pleasant and pretty sights for the patient's eyes to rest on—variety of objects, flowers, pictures*.

Florence Nightingale

## 1 Introduction

Good environmental design is regarded as a therapeutic resource for promoting health and well‐being and it is undeniable that the appearance and structure of hospitals matters to users [1, 2] with the earlier quote from Florence Nightingale in 1859 emphasising the impact and importance of fresh air spaces [3]. However, population growth and the explosion of healthcare treatments have led to high capacity and higher occupancy demands in healthcare buildings [4]. This requirement over the last century has impacted the built environment in which care is delivered [5], supplanting space that previously could have held nature areas to increase bed capacity.

The move from community healing spaces to large, high‐capacity buildings was first seen in the Victorian era after the industrial revolution. The effect of this has been well documented in architectural literature and health and building design [6], with many authors now calling for a more salutogenic approach [7]. That is, it is an approach that examines how individuals stay well, despite stress. There has been some research into this relating to long‐term patients and the influence of this approach in supporting patients with their mental health, their emotional processing of their illness and their ability to cope both during and after their hospital experience [8].

Understanding the impact of physical environmental stimuli in healthcare facilities allows the creation of environments that positively affect the healing process and impact the well‐being of patients [9]. A recent literature review of therapeutic hospital gardens in health care defined them as hospital gardens that are purposely designed and well integrated within the hospital grounds which medically, spatially and culturally support patient care and visitor and staff well‐being [10]. This is further reiterated in the evidence from the Intensive Care Unit (ICU) sphere of hospital design, with a recent literature review of the ICU built environment restating the importance of ICUs as a multiuser environment; functioning as a workplace for professionals, a place of care for patients and a place for connection with patient families and other relatives [11].

The evidence base for the exposure of the critically ill adult to therapeutic gardens is small and based largely on case studies [12, 13, 14, 15], clinical audit [16, 17], editorials, literature reviews of wider scope [11, 18, 19] and operational clinical guidance [20, 21]. There is some data on the impact of fresh air spaces for staff in ICU [22] and relatives in ICU [23] demonstrating that access to fresh air and gardens improves relatives' experience of the ICU environment and significantly mitigates the stress in family members of ICU patients [23].

The drive towards improving the evidence base for ICU patients and access to nature is in line with the concept of humanisation within the ICU environment. This theoretical framework is centred around the personalisation and optimisation of the patient and relative experience [24]. Recent work to explore the perceived stressors in ICU [25] found that environmental stressors like noise, sound, light and lack of privacy ranked highly from both patients, staff and loved ones. Steps that can be taken to mitigate these stressors are widely described in the literature [11] including nature‐based interventions [11, 26, 27, 28].

The objective of this case series is to explore the impact of ICU care delivered in an ICU Garden space. Two cases have been chosen to identify the opportunities for patients, relatives and staff to optimise the experience of hospitalisation and critical illness, including experiences at the end of life. Themes within these two cases will be presented, the clinical implications for practice and an exploration of the future opportunities for humanisation of critical care in line with the CARE guidance of reporting a case study [29].

## 2 Description of the Secret Garden

**FIGURE 1:**
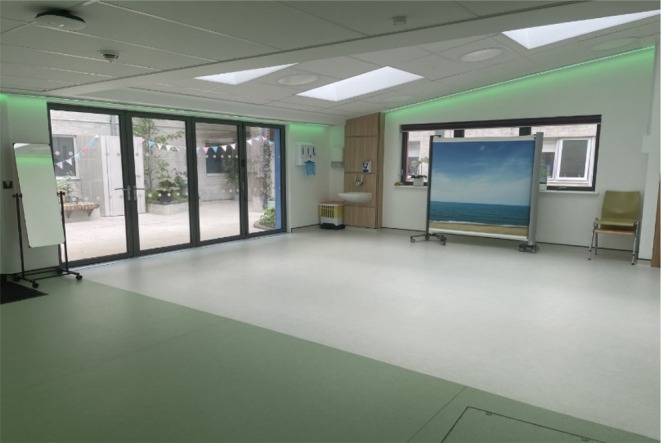
The Secret garden (Internal view).

**FIGURE 2:**
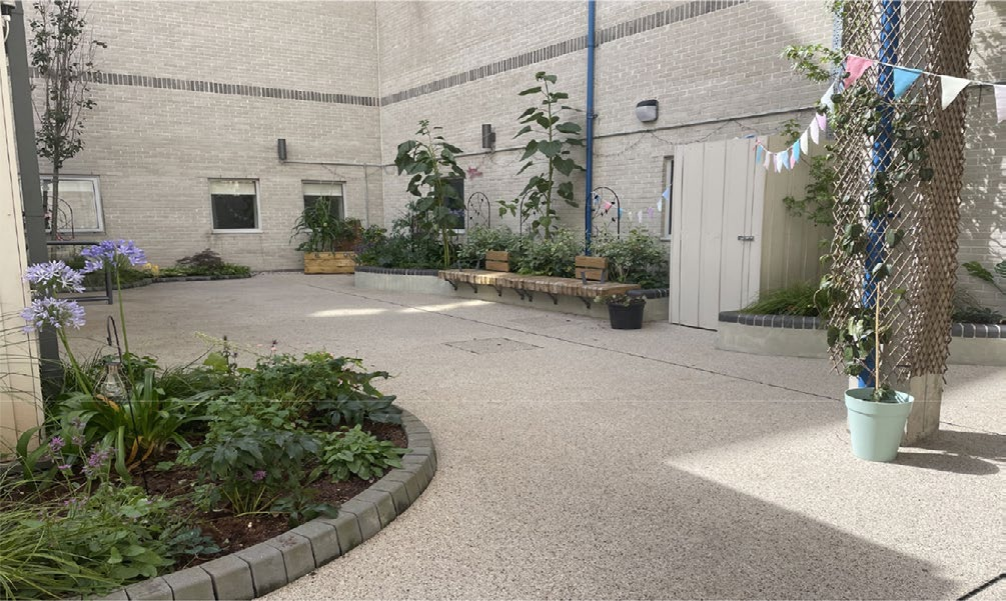
The Secret garden (external view).

The Secret Garden is a fresh air space, approx. 30 m × 40 m, in a courtyard located in an internal lightwell of a 1970s UK hospital. It has a rectangular internal building (Figure 1) that is air‐conditioned and heated, with bifold doors opening onto an L‐shaped garden with raised beds and trees. Internally, the space offers medical gases, monitoring, safety call bells, telephone, power and ventilation, handwashing, bespoke seasonal affective disorder lighting, sky lights and storage. Externally, there is a covered area, seating, multiple power sources, medical gases, suction and Wi‐Fi with the capacity to have 8–10 beds or chairs with visitors. The flooring is resin composite with attention to drainage, wheelchair and bed accessibility and safety. (See Figure 2).

## 3 Palliative Care in a Fresh Air Space

This ‘Secret Garden’ space has been utilised to support the palliative care of adults, paediatric patients, neonatal infants [15] and their loved ones. Utilising the Intensive Care Society national guidance [20], an adult case will be presented in this section and full consent from his relatives has been given.

## 4 Description of Case

The case presented is that of an adult male and their relatives' palliative care experience and death in the garden space. The case involved a planned withdrawal of ventilation after a short, unexpected 5‐day admission for pneumonia and respiratory support. The patient used the space for a 4‐h period after extubation and withdrawal from mandatory ventilation. At the time of his death, his family were able to be with him in the garden.

Two family members and the ICU nurse present during the end‐of‐life care and death provided feedback of their experience. A semi‐structured interview was completed with the two relatives; the interview was recorded, transcribed and thematically analysed by two researchers. This methodology allowed both detailed exploration of key themes, along with a flexible approach allowing participants to lead the discussion and uncover insight not initially anticipated by the research team.

**FIGURE 3:**
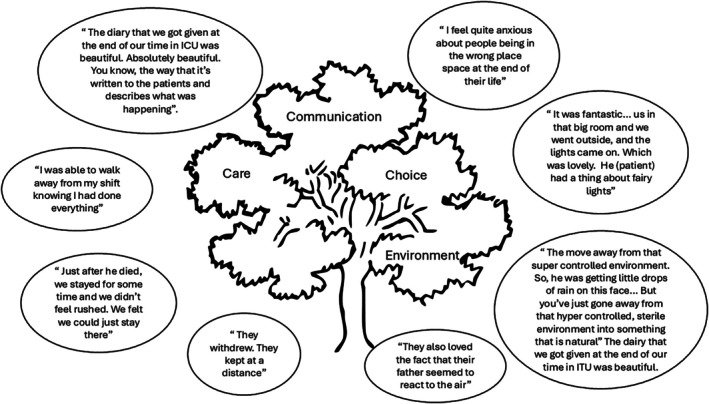
Themes from feedback at End‐of‐Life.

Written feedback/reflection was provided by the nursing team and this was analysed using content analysis. The data highlighted that despite the sadness of the end‐of‐life experience, the Intensive Care Garden space provided a positive environment for all respondents (Figure 3). Four main themes in the feedback emerged: environmental influence, communication and privacy, choice and personalisation, and care of the patient and relative.

### 4.1 Environmental Influences on End‐Of‐Life Care

The garden space appeared to offer a shift in the perception of the clinical environment, from acute and controlled to natural and less stressful. This shift has the potential to be positive or negative, as for some this shift could be challenging. The national guidelines explicitly mention safety and risk assessment and for this case the nursing team had two members of staff present to ensure adequate support both clinically and psychologically for the family and each other. The garden is also equipped with safety equipment (telephone) and care equipment (washing, sheets, hot water) in case of clinical need. This is to enable the minimisation of stress for the clinical team caring for the patient and their relative.

I feel quite anxious about people being in the wrong place at the end of their life (*Relative*).

### 4.2 Personalisation, Privacy and Choice

The garden is purposely private and has the capacity to screen off areas for complete privacy, whilst still allowing access to nature. This personalisation and privacy also extends to the garden room, with lighting, temperature, sound and screens. This supportively influences the perception of privacy within the space for patients, staff and relatives.

They withdrew, they kept at a distance. (*Relative* speaking about nursing staff)

### 4.3 Communication and Privacy at End‐Of‐Life

The nursing team at the bedspace first mentioned the possibility of the family using the garden for end‐of‐life care, and this was explicitly mentioned by the family as an important connection for them. This allowed the nursing team to lead the care outside and to optimise their perception of their ability to provide positive care experiences at end‐of‐life. The nursing team were present during the end‐of‐life care but at a distance to ensure privacy for the family; this was positively received by the family. The garden space allowed a larger number of relatives to be present as it is approximately six times the size of a standard ICU bed space.

It was fantastic…us in that big room and we went outside, and the lights came on, which was lovely. He (patient) had a thing about fairy lights. (*Relative*).

### 4.4 Perceptions of Increased Care and Compassion

The combination of a less clinical environment with greater levels of privacy and personalisation was reported by the family to increase their perception of a clinical team acting with care and compassion. Although the clinical team was small (2 nursing staff), it was perceived as hugely supportive and caring.

I was able to walk away form my shift knowing that I had done everything (*Nurse*).

This echoes the wider evidence base in the environmental influences on palliative care which emphasises the importance and impact of environment, privacy, dignity, personalisation, family contact and communication [30]. Another recent case study from this garden space, with a neonatal patient, echoed these findings [15].

This case highlights that end‐of‐life care in a fresh air space in ICU is feasible and supports relatives to report a positive ‘natural’ experience of death. End‐of‐life care in the fresh air in an inpatient hospital setting is rare, and in ICU is not widely described in the literature. This is a unique case describing the impact of an Intensive Care Garden space on relatives' experience at end‐of‐life and forms part of a larger project exploring end‐of‐life care in hospital gardens.

## 5 Rehabilitation in a Fresh Air Space for Patients With Hospital‐Acquired Infection

### 5.1 Description of Case

An adult case will be presented in this section, and full consent has been given. A previously fit and well 59‐year‐old male, with a past medical history of Marfan syndrome and tinnitus, was on holiday abroad when he was diagnosed with a Sub Arachnoid Haemorrhage. The haemorrhage was exerting pressure effect on his brainstem, and he was treated with a right vertebral artery coiling. Whilst in ICU, he had an extra ventricular drain fitted, was fully ventilated, and required a tracheostomy for respiratory and bulbar management. He was repatriated to his home ICU in England 6 weeks post ICU admission, and no rehabilitation had been initiated at this point. He presented to ICU in the UK globally weak, withdrawn, non‐communicative, hypoactive delirium, ventilated and infected/colonised with multiple multi‐drug‐resistant organisms (MDRO). We are pleased to report that after prolonged care in an inpatient setting this gentleman was discharged and is now having support from rehabilitation services. This case report describes the care received in the garden space whilst in ICU for 3 months after re‐patriation to the United Kingdom.

**FIGURE 4:**
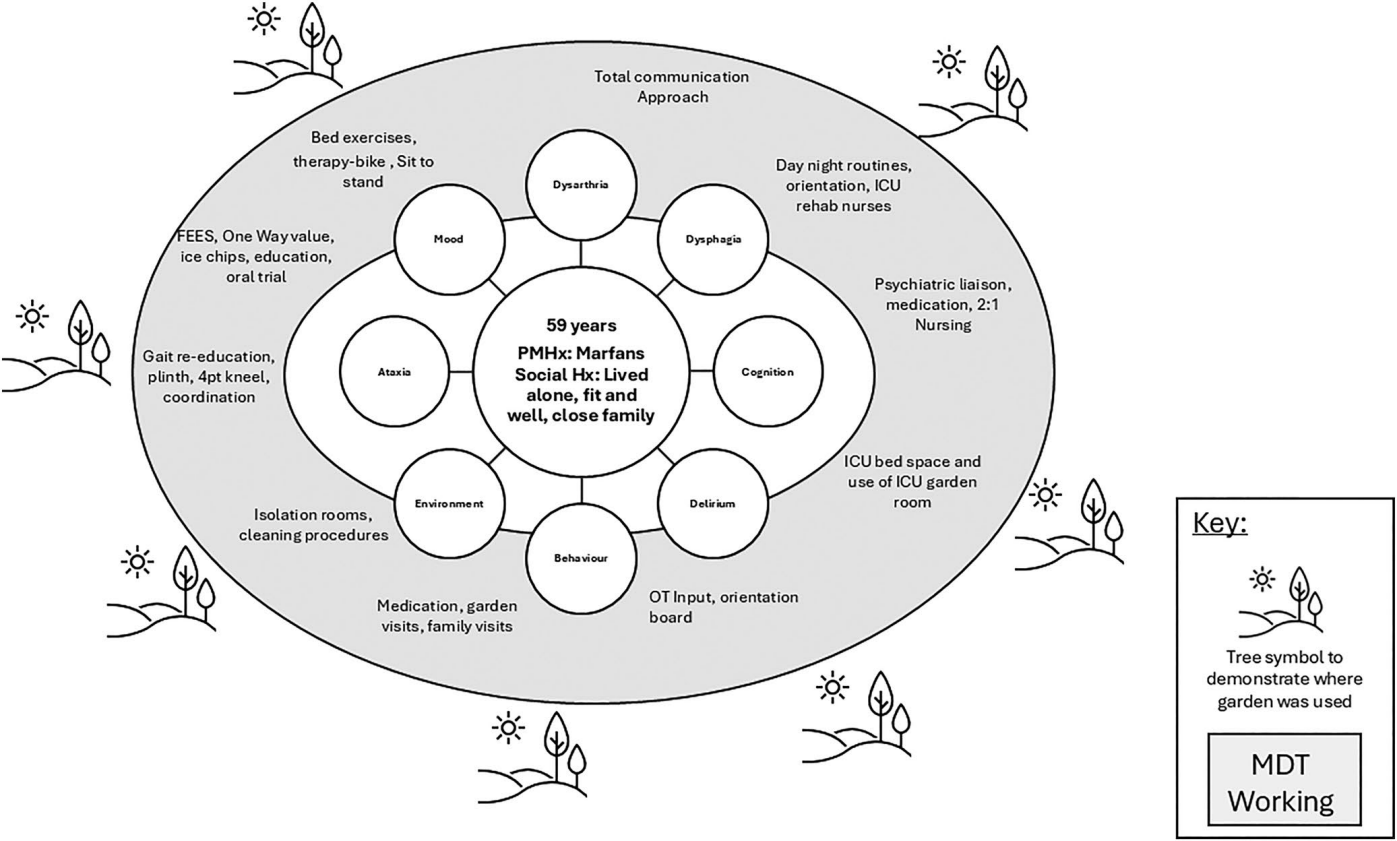
Patient‐specific impact of ICU garden.

Multiple factors influenced his rehabilitation and weaning from mandatory ventilation as he had numerous complex rehabilitation needs. Specifically, he was still suffering from high levels of delirium, alongside dysarthria, dysphagia, cognitive impairment, mood and behavioural disturbances (related to injury and a long period of time away from relatives), skeletal muscle loss and ataxia (Figure 4). Strict locally enforced infection prevention control (IPC) measures limited the amount of rehabilitation equipment that the patient could access in our normal rehabilitation spaces. This limited his capacity to access rehabilitation equipment. The ICU Garden and associated ICU garden room were therefore utilised as a bespoke rehabilitation space (see images 1 and 2) with strict IPC measures put in place. The case study is a presentation of the care delivered in the garden space and its associated room.

### 5.2 Functional Rehabilitation Space

The rehabilitation garden not only provided a functional rehabilitation space but also presented the MDT with some unique challenges. Due to the garden's distance from the clinical areas (150 m), extra and adequately airway trained staff were needed to ensure patient and staff safety. Furthermore, there was a limited availability of teams due to the strict IPC procedures. Rehabilitation sessions were planned for the end of the day to eliminate the possibility of spread of the MDRO to any other patients, and to allow the full deep clean of the environment thereafter. The garden room is a large empty space (see Image 1) and therefore lacked the specialist rehabilitation equipment that would be found in a specialised neurological physiotherapy gym. Staff had to adapt and use easily moveable and cleanable equipment, such as the Arjo Walker, plinth and floor mats, in novel ways.

### 5.3 Environmental Optimisation

This garden space allowed optimisation of environmental conditions, promoting an appropriate day–night routine, rebalancing circadian rhythms, facilitating sleep and supporting family interactions, in a quiet and less stimulating environment than the ICU. We found this to be effective when managing behaviour, delirium, cognitive engagement and mobilisation. The garden room (Image 1) is fitted with seasonal affective disorder lights optimising the level of luminous flux per unit area (LUX) that the patient was exposed to, whilst also having access to floor‐to‐ceiling windows and the ability to do rehabilitation in the fresh air. Cognitive engagement, mood and delirium were significantly improved following garden rehabilitation sessions.

### 5.4 Delirium Management

For the prevention and treatment of delirium, non‐pharmacological treatments such as early mobilisation are recommended [31], and we were aware that rehabilitation had the potential to reduce the overall duration of this patient's delirium so needed to be prioritised. The novel utilisation of an ICU garden space allowed us to achieve timely rehabilitation goals, cluster care, limit environmental stressors such as noise and artificial light, and optimise patient experience with relatives. As disturbed circadian rhythm is potentially a modifiable cause of delirium among patients in ICU [32] we were keen to utilise the bright light therapy in our garden room as part of a multi‐component delirium management programme. This approach has been validated in older hospitalised adults [33] and has recently been found to be impactful in recent work [17, 34, 35].

### 5.5 Environmental Optimisation

MDROs bring new and complex challenges to rehabilitation and often impact rehabilitation care negatively, with strict IPC constraints on both equipment and staffing capacity [36]. The literature describes the negative impact of MDROs on patients and relatives, who describe feelings of unworthiness; fears and worries of varying intensity; lingering unanswered questions; confusion due to discrepancies in MDT behaviours and hospital policies; stress due to precautionary measures and other regulations surrounding MDRO carriage; disruptions to other medical conditions; and the perception of carrying the burden of responsibility for the MDRO on their own shoulders [36]. Environmental isolation of patients therefore needs to be managed carefully with a supportive and informative approach from clinical teams. The garden room in this case study was utilised as a supportive strategy for environmental enrichment, with relatives encouraged to attend. Feedback was positive from the patient's relatives, but no formal exploration took place, so we were unable to assess whether there were any negative perceptions, such as whether the garden room utilisation added to their burden of isolation. No formal written feedback was taken from staff, patients or relatives, and this is recognised as a limitation of this case study.

### 5.6 Management of Infection Control

Compliance with environmental cleaning for the immediate surrounding area is a key recommendation to decrease infection caused by MDROs [37], so support from IPC and domestic technical teams was key to ensure patient and staff safety. Specialist cleaning regimes were introduced to reduce the risk of MDRO transmission. Support for clinical staff when providing care in clinical environments with increased levels of personal protective equipment was reminiscent of Covid‐19 so care was taken to ensure preparation for transfer and extra clinical staff where available.

## 6 Summary for Clinical Practice

Feasible—End‐of‐life care and rehabilitation in fresh air spaces is feasible in ICU environments.

Supportive—Fresh air spaces offer end‐of‐life care in a more natural and less clinical environment.

Management of risk—Preparation is key to ensure everyone involved in the transfer is supported and patient care is not impacted negatively.

Personalisation of environment—Fresh air spaces can offer patient centred flexible and responsive environments with greater privacy to care for the needs of patients and relatives.

Lack of clinical research—Further research is needed to confirm the benefits of fresh air therapy for the intensive care patient.

## 7 Conclusions

Across all healthcare environments views of nature and/or gardens are reported to increase levels of positive feelings, with access to outside space deemed to be extremely valuable for patients, carers, relatives and staff [4, 22, 23, 38]. Currently, there is no large‐scale evidence of the impact of access to nature for ICU patients, and this impacts the adoption in practice. A factor that may inhibit the research of ICU patients utilising nature spaces is the issue of risk and safety when moving critically ill patients out of the ICU environment. Safety and management of risk are represented in the literature with recent work on the safety events of mobilisation to a fresh air space [13, 16]. National guidance has been written to support and optimise patient safety issues related to transfer to fresh air environments [21] but it remains a key barrier in clinical practice. Safety events have been established in studies on early mobilisation [39, 40, 41, 42] but no large‐scale, multi‐centre studies have been done in the ICU population to establish the risk versus benefit of accessing fresh air spaces.

This case series has demonstrated two examples of the utilisation of a fresh‐air space for patients in ICU. These cases have limited methodological rigour and are not able to be used for wide scale inference; however, in both cases, garden use was found to be safe, feasible and optimised the experience of the patient, relatives and staff. Confounding variables such as sound, light, privacy, open visiting and single room ICUs could also be related to the views expressed here.

Personalisation of the ICU environment is a key intervention to optimise patient, relative and staff experience and can be implemented in many different ICU formats. This is echoed in the move towards supporting humanisation of the ICU environment and recent GPICS guidance. It is hoped that this paper offers the opportunity to explore the clinical challenges and report the benefits of accessing nature—whilst personalising and humanising the ICU experience.

## Conflicts of Interest

The authors declare no conflicts of interest.

## Data Availability

Research data are not shared.
